# Comprehending the dynamism of B chromosomes in their journey towards becoming unselfish

**DOI:** 10.3389/fcell.2022.1072716

**Published:** 2023-01-04

**Authors:** Vijay Rani Rajpal, Suman Sharma, Deepmala Sehgal, Prashansa Sharma, Nikita Wadhwa, Priyanka Dhakate, Atika Chandra, Rakesh Kr. Thakur, Sohini Deb, Satyawada Rama Rao, Bilal Ahmad Mir, Soom Nath Raina

**Affiliations:** ^1^ Department of Botany, Hansraj College, University of Delhi, Delhi, India; ^2^ Department of Botany, Ramjas College, University of Delhi, Delhi, India; ^3^ Syngenta, International Maize and Wheat Improvement Center (CIMMYT), Texcoco, Mexico; ^4^ University School of Biotechnology, Guru Gobind Singh Indraprastha University, New Delhi, India; ^5^ National Institute of Plant Genome Research, New Delhi, India; ^6^ Department of Botany, Maitreyi College, University of Delhi, New Delhi, India; ^7^ Amity Institute of Biotechnology, Amity University, Noida, Uttar Pradesh, India; ^8^ Department of Biotechnology and Bioinformatics, North Eastern Hill University, Shillong, Meghalaya, India; ^9^ Department of Botany, University of Kashmir, Srinagar, India

**Keywords:** B chromosomes, non-mendelian transmission, genetic drive, non-disjunction, preferential fertilization, adaptive significance

## Abstract

Investigated for more than a century now, B chromosomes (Bs) research has come a long way from Bs being considered parasitic or neutral to becoming unselfish and bringing benefits to their hosts. B chromosomes exist as accessory chromosomes along with the standard A chromosomes (As) across eukaryotic taxa. Represented singly or in multiple copies, B chromosomes are largely heterochromatic but also contain euchromatic and organellar segments. Although B chromosomes are derived entities, they follow their species-specific evolutionary pattern. B chromosomes fail to pair with the standard chromosomes during meiosis and vary in their number, size, composition and structure across taxa and ensure their successful transmission through non-mendelian mechanisms like mitotic, pre-meiotic, meiotic or post-meiotic drives, unique non-disjunction, self-pairing or even imparting benefits to the host when they lack drive. B chromosomes have been associated with cellular processes like sex determination, pathogenicity, resistance to pathogens, phenotypic effects, and differential gene expression. With the advancements in B-omics research, novel insights have been gleaned on their functions, some of which have been associated with the regulation of gene expression of A chromosomes through increased expression of miRNAs or differential expression of transposable elements located on them. The next-generation sequencing and emerging technologies will further likely unravel the cellular, molecular and functional behaviour of these enigmatic entities. Amidst the extensive fluidity shown by B chromosomes in their structural and functional attributes, we perceive that the existence and survival of B chromosomes in the populations most likely seem to be a trade-off between the drive efficiency and adaptive significance versus their adverse effects on reproduction.

## 1 Introduction

B chromosomes (Bs) exist as accessory entities in addition to the standard chromosome complements (As) in more than 3,000 species of plants, animals and fungi (Jones and Rees, 1982; Houben et al., 2014; Houben, 2017; D’Ambrosio et al., 2017; Jones, 2018; Chen et al., 2022). Though dispensable, they maintain themselves in an accumulative manner in various tissues of an organism and subsets of populations (Douglas and Birchler, 2017; Ahmad and Martins, 2019; Ahmad et al., 2020; Houben et al., 2021). It is generally assumed that Bs are generated from As through various sequence accumulation and reorganization processes. The sequence accumulation occurs through the amplification of certain families of repetitive sequences and transposable elements (Lamb et al., 2007) and leads to intra- and interspecific genetic variation making them an interesting genomic model to investigate evolutionary processes (Vujošević et al., 2018).

Bs represent a classic work of evolution and possess many unique characteristic features. These supernumerary chromosomes do not pair with the As of their hosts during meiosis and vary in their number, size, composition, structure and segregation pattern across taxa (Banaei-Moghaddam et al., 2015). Bs are not always present in pairs and do not follow Mendelian segregation principles. The centromeres in chromosomes represent a region of highly specialized repetitive chromatin and along with the pericentromeric heterochromatin regulate accurate segregation during cell division across eukaryotes (Yamagishi et al., 2008). It is speculated that the centromere of B evolved from As and then differentiated from the latter by an accumulation of additional B-specific centromeric and pericentromeric repeats/or specific retrotransposons and involvement of rearrangements as seen in rye, maize and other organisms (Jin et al., 2005; Banaei-Moghaddam et al., 2012).

Bs show irregular meiotic behaviour and ensure their successful transmission through non-Mendelian inheritance mechanism called chromosome drive involving unique non-disjunction process (Houben et al., 2014; Houben, 2017; Jones, 2018). The chromosome drive refers to the transmission advantage that results from a higher than 0.5 rate of chromosomes’ transmission to the next generation, disregarding the Mendelian law of equal segregation of maternal and paternal chromosomes. The most likely factor propelling the drive is the phenomenon of non-disjunction, a centromeric activity that refers to the failure of homologous chromosomes or sister chromatids to separate accurately during cell division most likely caused due to their extended cohesion. It results in passage of two copies of Bs in a single gamete accounting for the Bs’ accumulation mechanism (Banaei-Moghaddam et al., 2012). In most species of plants, fungi and animals, where chromosome drive has been demonstrated to be the key mechanism of Bs’ transmission, it has been reported that drive occurs during pre-meiotic, meiotic or post-meiotic divisions. In the family poaceae, for example, the drive is mostly post-meiotic, occurring in the first or second pollen grain mitosis. Maize, rye and goat grass among plants, *Magnaporthe oryzae*, *Zymoseptoria tritici* and *Nectria hematococca* among fungi and grasshoppers and the mealybug *Pseudococcus obscurus* among animals represent the best examples wherein the drive mechanisms have largely been explored (Jones 2018; Camacho, 2022). Besides drive, mitotic and/or meiotic instability could also lead to the non-Mendelian inheritance of Bs.

The B chromosome research has come into the limelight during the past two decades. The advancements in molecular cytogenetics, genomics, and transcriptomics technologies have successfully unveiled the B-associated enigmatic processes, although a lot needs to be done to unearth the mechanistic behavioural aspects associated with their differential presence, transmission, and sustenance in populations. Further, the availability of reference genome sequence from several eukaryotic species along with improved “B omics” platforms have opened vistas to zoom into genes and coding sequences located on the Bs (Ahmad and Martins, 2019). These developments have greatly enhanced our understanding of the structural, functional, and evolutionary relevance of Bs (Huang et al., 2016; Makunin et al., 2016; Ma et al., 2017). In this review, we have consolidated the published information to highlight and understand the evolutionary changes that have occurred in the structure, composition, inheritance, behaviour and functionality of Bs in plants, fungi, and animals in order to explore their journey from selfishness to becoming unselfish for their survival.

## 2 Occurrence

Since their first discovery in *Metapodius* (now called *Acanthocephala*), the leaf-footed plant bug insect, more than a century ago (Wilson, 1907a, Wilson, 1907b), B chromosome research has received immense attention. Bs were reported in plant species (rye and maize initially) in the 1920s and 1950s (Gotoh, 1924; Kuwada, 1925; Longley, 1927; Darlington and Wylie, 1956), in mammals (marsupial glider and fox) in 1960s (Hayman and Martin, 1965; Moore and Elder, 1965) and in locusts (Kayano, 1971; Lespinasse, 1977) in 1970s. Since then, a plethora of reports has constantly enhanced our knowledge regarding the distribution of Bs across life forms (Burt and Trivers, 2006; Douglas and Birchler, 2017; Ahmad et al., 2020; Houben et al., 2021).

Darlington and Wylie (1956) investigated the number of chromosomes in over 17,000 species of flowering plants and reported that 0.8% of flowering plant species have Bs. Later, the occurrence of Bs in∼3% of eudicots and ∼8% of monocots was reported by Levin et al. (2005). Most recently, D’Ambrosio et al. (2017) compiled reports of Bs published from 1907 to 2016 from monocots, eudicots, gymnosperms, fungi, insects, fish, and mammals in an online database called “B-chrom!”. This database contains the most updated information on Bs number and ploidy levels of 1,095 genera belonging to 311 families (185 families of animals, 119 families of plants, and seven families of fungi). Some taxonomic orders show an exceptionally high percentage of Bs such as Asparagales (15.72%), Asterales (20.75%), Poales (21.76%), Commelinales (27.2%) in plants (Jones et al., 2008; D’Ambrosio et al., 2017) and order Orthoptera (Camacho, 2005) in animals with hotspot superfamilies Acridea (14.6%), Grylloidea (14.9%), Pyrgomorphoidea (32.3%) and Tetrigoidea (14.3%) (Palestis et al., 2010).

Bs are reported to contribute significantly to intraspecific variation in nuclear DNA amounts being an underlying factor for DNA variation in natural populations in plants and animals (Camacho et al., 2000; Martins et al., 2014; Jones, 2018; Komissarov et al., 2018). Numerically, Bs range from one to a few univalents (Camacho, 2005; Dhar et al., 2017) to more than 30 in both plants and animals. Among plants, *Pachyphytum fittkaui* (Crassulaceae) has recorded the highest (50) number (Uhl and Moran, 1973), followed by *Albuca bracteata* (Asparagaceae) and *Zea mays* with 36 and 34 Bs, respectively (D’Ambrosio et al., 2017). Among animals, the rodent *Apodemus peninsulae* (Muridae) possesses the highest B chromosomes (30) followed by the spider *Clubiona japonicola* (Clubionidae) (D’Ambrosio et al., 2017), the wood mouse (*Apodemus peninsulae*) (Volobuev and Timina, 1980) and the fly, *Xylota nemorum* (Boyes and van Brink, 1967) with 28, 24 and 24 Bs, respectively.

The size of Bs is highly variable ranging from a dot micro Bs to macro or mega Bs. Usually, Bs are smaller than A chromosomes but in some animal species such as cyprinid fish (Ziegler et al., 2003; Schmid et al., 2006), the characid fish (Mestriner et al., 2000) and the giant white-tailed rats (Baverstock et al., 1982), Bs bigger than the largest A chromosomes have been reported. B chromosome with 29.7% of the length of the A set and even more than twice the size of the A chromosome have been reported in Korean field mice *Apodemus peninsulae* (Borisov and Muratova, 2010) and *Plantago lagopus* (Dhar et al., 2002) respectively. Generally, the micro-Bs are thought to be generated from breaks in the A set and they further re-organize to form the macro-Bs (Borisov and Muratova, 2010).

Bs are not ubiquitously present in all members of a population or all species of a genus. Numerous B chromosome variants have been observed in *Aegilops speltoides* genotypes (Shams and Raskina, 2020). In some eukaryotes, they have been observed only in the generative tissues (Jones and Rees, 1982). Even within the same organism, Bs are not necessarily present in all the cells and even show somatic instability in many species. For instance, numerical differences in the Bs were observed in roots and the claudicle leaves in *Crepis capillaris* (Rutishauser and Rothlisberger, 1966). Likewise, differential preservation of Bs in the primary roots and their absence in the adventitious roots has been recorded in both *Agropyron cristatum* and *Poa alpina* (Müntzing and Nygren, 1955; Baenziger, 1962). Further, in a few species namely *P. timoleontis* (Nygren, 1957), *Haplopappus gracilis* (Ostergren and Frost, 1962), *Aegilops* species (Mendelson and Zohary, 1972) and *S. purpureosericeum* (Janaki Ammal, 1940; Darlington and Thomas, 1941), Bs show a preferential elimination in roots. Due to the irregular chromosome elimination, *Sorghum* species show mosaicism in the distribution of Bs. While Bs are unstable in young shoots, ovaries, and tapetal cells, and are showcased only in the fertile florets in *S. purpureosericeum*, (Janaki Ammal, 1940; Darlington and Thomas, 1941), they are completely eliminated from root tissue in *S. nitidum and S. halepense*. Similarly, they have been eliminated in root, stem and meristem cells in *S. stipoideum*, while being mosaically present in microsporocytes and tapetal cells (Bednářová et al., 2021). Such chromosome elimination accompanying cell differentiation is a unique feature associated with Bs and has been first documented in *S. purpureosericeum* among plant species (Karafiátová et al., 2021), although elimination of Bs from somatic cells of adults has previously been reported from the *Polycelis tenuis* (flatworm) (Melander, 1950) and *Leptothorax spinosior*, the myricine ant (Imai, 1974)*.* The B chromosomes are stable only in the germ line of males in the latter. Bs are totally eliminated in the roots upon embryo differentiation and are just retained in the aerial parts in rye. The chromosomal elimination in the roots is considered a result of the non-disjunction of B chromatids and is underpinned as a mechanism of differential maintenance of Bs in the shoots as the B-located root-specific gene expression might be harmful to normal plant development (Ruban et al., 2020). Recently, Li et al. (2022) obtained different types of restructured Bs by X-ray mutagenesis to study the chromosome elimination process restricted in roots of rye and found standard wild-type Bs in the shoots of all mutagenized plants. Besides, 40 B variants were identified that inconsistently escaped the root elimination. Most importantly, the B-A translocations that contained a small fragment of B attached to A chromosome (with A centromere) could only sustain stably in shoots as well as roots and escaped the elimination process. Root cells containing B-derived centromere conspicuously did not possess any B-A translocation chromosomes, clearly implying that the B centromere played a key role in the elimination process of chromosomes (Li et al., 2022). The above examples explicitly indicate that Bs have devised dynamic strategies to secure their sustenance and perpetuation through various unusual mechanisms.

## 3 Structural organization and composition of Bs

The structural organization of Bs has been extensively investigated through molecular cytogenetics, microdissection, or flow sorting approaches (Silva et al., 2014). More recently, the availability of several completely sequenced eukaryotic genomes and the advances in the next-generation sequencing (NGS) platforms have provided unprecedented opportunities to zoom into Bs in a wide variety of life forms (Ahmad and Martins, 2019), leading to enhanced understanding of the dynamic changes in the structure and functions of Bs (Ahmad et al., 2020; Blavet et al., 2021).

Structurally, Bs can be acrocentric (single long arm), metacentric (bi-armed) with two equal arms, or submetacentric (bi-armed) with a short and a long arm connected *via* the B-centromere (BC) (Moraes et al., 2007). It is common to encounter diversity in forms of Bs within a single species. Two types of B chromosomes were observed in sorghums, including a short B (S) of a size similar to the A chromosome and a two times larger isochromosome (L) (Reddy, 1958). Similarly, in *Aegilops mutica,* both meta- and telocentric Bs were observed (Mochizuki, 1960). Further, in rye six different forms of Bs were found (Jones and Rees, 1982) while in *A. schoenoprasum,* the highest, 29 forms existed (Bougourd and Parker, 1979). Palestis et al. (2004, 2010) reported the prevalence of Bs in karyotypes with acrocentric A chromosomes in both mammals and orthopteran insects.

Bs have shown a great intra- and inter-specific diversity in the proportion of euchromatic and heterochromatic regions. Ranucci et al. (2021) showed that Bs in two species of fish, *Moenkhausia bonita* and its allopatric species *M. forestii*, vary in the relative position as well as the content of the heterochromatin. In *M. bonita*, there is little heterochomatin found at the terminal and peri-centromeric regions and in *M. forestii*, on the other hand, extensive heterochromatic blocks are present in the interstitial regions. In plants, rye and maize have been used extensively as model systems to dissect the structure of Bs and to understand the sequence complexity. Most significantly, Bs have been revealed to be a mosaic of organellar and nuclear genomes (Martis et al., 2012), having telomeric and centromeric domains sharing homology with the domains on the A chromosomes (Jones, 2012).

Large amounts of heterochromatin has been involved in the structural composition of Bs. Lima-de-Faria (1962) reported two sequence families, D1100 and E3900, in the sub telomeric domain of the long arm of the B chromosome. Later, Endo et al. (2008) conducted a comprehensive behavioral analysis of chromosomes in wheat lines carrying introgressed fragments of rye Bs and concluded that D1100 and E3900 sequence families remain highly conserved in their organization (Niwa and Sakamoto, 1995; Marques et al., 2013). Wilkes et al. (1995) using fluorescent ISH showed that D1100 preferentially accumulates in two zones in the sub telomeric region, while E3900 has a distal and more homogeneous signal, which overlaps with the domain D1100. Investigations conducted on copy number variation of E3900 sequences on B and A chromosomes in other cereals including rye, A genomes have revealed high copy numbers of 100–150 on Bs and low copy numbers on A chromosomes (Pereira et al., 2009). Similar, centromeric CentC repeats and pericentromeric ScC111 repeats have been located in maize and rye, respectively (Jin et al., 2005; Banaei-Moghaddam et al., 2012).

Another important repeat sequence tandemly present in Bs is satDNA, usually distributed particularly at pericentromeric, sub telomeric and interstitial positions in the chromosome. In *Locusta migratoria* (Ruiz-Ruano et al., 2018), five satellite DNAs, located on autosome nine were also observed in the Bs. satDNA LmiSat 02-176 of these repeats’ accounts for 54.8% of B chromosome DNA (Camacho et al., 2000; Camacho, 2005; Ruiz-Ruano et al., 2018). Similar findings have been reported for grasshopper *E. plorans* (Montiel et al., 2012).

In maize, extensive research has been conducted on the B-specific repeat sequence and it has been reported that it is very similar in sequence to the heterochromatin knob present in the normal A chromosomes (Jin et al., 2005). Knobs have a high copy number of repeated units that stain deeply with chromatin specific stains. Two types of knobs have been discovered in maize; the 180 and 350 bp unit lengths knobs. The 180 bp knob consisting of 180 base pair tandem repeats, was first recognized by Peacock et al. (1981). These were largely located at interstitial regions on maize chromosomes (Buckler IV et al., 1999). The 350 bp knob, also called the TR1 repeat, consists of 350 base pairs tandem repeats, and is highly related to the 180 bp repeat at the sequence level (Ananiev et al., 1998). Further, sequence analysis and mapping have also shown that *Copia* and *Gypsy* are the most frequent transposable elements (TEs) found in maize landraces as well as cultivars. Interestingly, for the reasons not understood well, in landraces, the upstream and downstream of genic sequences harbour TEs, while in cultivars TEs are present in the intergenic regions (Schnable et al., 2009; Sun et al., 2018).

A completely heterochromatic B chromosome is reported by Dhar et al. (2002) in *Plantago lagopus*. Later, Kour et al. (2013), using reverse genomic *in situ* hybridization (GISH) and fluorescence *in situ* hybridization (FISH) techniques, also showed localization of 5S rDNA on the entire B chromosome suggesting that the entire body mass of the B chromosome is derived from 5S rDNA sequences while the 45S rDNA sequences were localized only below the telomeric sequences. Recently, Kumke et al. (2016) analysed the DNA composition in *P. lagopus B+ and B-* plants, using cutting-edge sequencing technology and reported that the nuclear genome of *P. lagopus* is rich in repetitive sequences (68% of the genome) with the Maximus/SIRE lineage of Ty1/Copia LTR-retrotransposons as the dominant repeat type representing 25% of the genome. Similarly, many ribosomal DNA loci are organized on Bs as arrays of rDNA satellite repeats. For instance, in both the plant species *C. capillaris* and in grasshopper *E. plorans,* genes are transcribed as long heterogeneous precursor 45S rDNA (constituting 18S, 5.8S and 28S) while in *Brachycombe dichromosomatica* no 45S rDNA is transcribed (Marschner et al., 2007). Anjos et al. (2016), reported the presence of two distinct Bs in the grasshopper *Eumastusia koebelei* that showed the presence of 18S rDNA, U1 snDNA and U2 snDNA similar to the sequences present in the autosomes.

**FIGURE 1:**
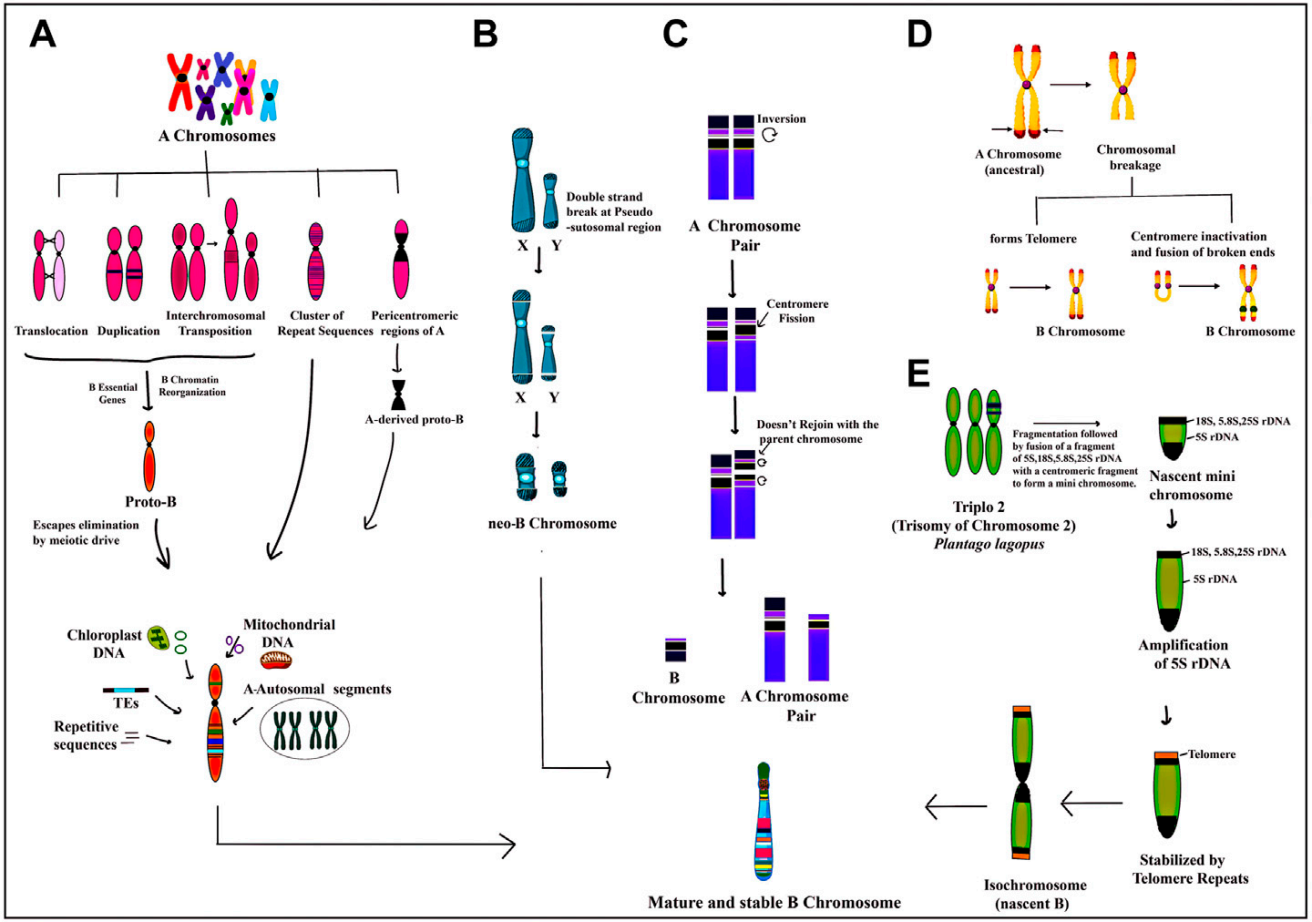
Depiction of various proposed models on the origin of B chromosomes:**(A)** genomic DNA amalgamated from multiple A chromosomes undergo structural rearrangements like translocation, duplication, transposition, insertion of repeat sequences and pericentromeric regions followed by the addition of a few B essential genes required for their successful transmission and chromatin reorganizing genes resulting in the formation of a nascent Proto-B chromosome. The Proto-B chromosome is further modified by the addition of transposable elements and DNA sequences from the organellar genome and ampliconic multiple A chromosome sequences to form a mature stable B chromosome with diverse functions. **(B)** the pseudoautosomal region (PAR) on X and Y chromosomes undergoes double-stranded breaks which in association with the centromere leads to the formation of neo-B chromosome. **(C)** One homologue of the A chromosome pair which has a secondary constriction on its short arm undergoes inversion and centromeric fission. The fission portion of the short arm inverts again without joining back at the same position resulting in the formation of an A chromosome without secondary constriction and a newly formed B chromosome which is further stabilized by the incorporation of many other types of DNA fragments. **(D)** ancestral A chromosome has a few hotspot regions which undergo breakage forming a chromosome without a telomeric end. This broken chromosome can either form a new telomeric end or the broken arms of the chromosome fuse following the inactivation of the centromere. Both mechanisms finally result in the formation of B chromosome **(E)** triplo 2 is a translocation trisome of chromosome 2 in *Plantago lagopus* in which rDNA regions 18S, 5.8S, 25S and 5S undergo breakage, and fragmentation followed by fusion with centromeric fragments to form a nascent mini B chromosome. Subsequently, there is selective amplification of 5S region along with the addition of telomeric repeats followed by misdivision of centromere leading to the formation of an iso-chromosome type of B.

The segments of organellar genomes and functional genic fragments from the set of standard chromosomes have contributed to the formation of Bs in various taxa (Valente et al., 2014; Ruban et al., 2017; Marques et al., 2018; Blavet et al., 2021; Pokorná and Reifova, 2021). In rye, for example, the B chromosome is shown to be a fusion product of the A chromosome and chloroplast- and mitochondria-derived sequences (Martis et al., 2012). The NGS sequencing of Bs across taxa has confirmed Bs to be largely composed of A paralogues and added organellar segments (Valente et al., 2014; Ruban et al., 2017; Marques et al., 2018; Blavet et al., 2021; Pokorná and Reifova, 2021). The genetic consequences of the accumulation of organellar DNA on Bs are less deleterious than their counterparts on As, wherein insertions may disrupt gene functioning leading to serious consequences, especially in homozygous conditions. The various propositions put forth on the structure of Bs in various taxa have been developed and consolidated in Figure 1.

In addition to euchromatic functional genes and heterochromatic repetitive sequences, a few pseudogene like sequences have been identified on Bs (Banaei-Moghaddam et al., 2013; Makunin et al., 2018; Kichigin et al., 2019). Chromosomal rearrangements and sequence duplications in As have been held responsible for origin of Bs (Banaei-Moghaddam et al., 2015) in Korean field mice (Karamysheva et al., 2002; Rubtsov et al., 2009) grey brocket and Siberian roe deer (Makunin et al., 2016).

## 4 Origin and evolution of Bs

Extensive research has been done to understand the origin and evolution of Bs that still remains an enigma. Various theories put forth propound Bs origin either from sex chromosomes or autosomes (A set) of either the same species or from a related species as a result of interspecific hybridization (Lopez-Leon et al., 1993; López-León et al., 1994; Camacho et al., 2000; Perfectti and Werren, 2001; Cabrero et al., 2003; Utsunomia et al., 2016). Other propositions include deletion of translocation trisomes (Wiebe et al., 1974), chromosome fragmentation (Peeters et al., 1985), a fusion of different chromosomes (Martis et al., 2012; Silva et al., 2014; Valente et al., 2014) or the fusion product of A chromosome along with organellar sequences (Martis et al., 2012). They have also been shown to originate from the heterochromatin pericentromeric region of the sex chromosomes (Ventura et al., 2015; Rajičić et al., 2017) or from heterochromatin of both autosomal and sex chromosome (Rubtsov et al., 2009). The pseudoautosomal region (PAR) on X and Y chromosomes, which pairs them in the pachytene stage of meiosis undergoes double-stranded breaks which together with the centromere, makes this region of the sex chromosomes more suitable for neo-B formation (Figure 1) (Rajičić et al., 2017). Unequal crossing over of Bs may also produce some variants of B chromosome (Cheng et al., 2016). Various molecular cytogenetical studies in animals have provided experimental evidence for the interspecific origin of Bs. For instance, Perfectti and Werren (2001), who observed that centric fragments were generated from chromosome breakage during the interspecific transfer of chromosomes from *Nasonia giraulti* into *N. vitripennis*. The authors concluded that interspecific hybridization leading to unstable centric fragments can provide impetus to B chromosome generation. Figure 1 collates all the possible mechanisms that have been postulated to explain the origin of Bs.

Extensive research has been done in rye (Jones and Rees, 1982; Puertas et al., 1985; Puertas et al., 1998; Marques et al., 2012; Houben, 2017; Houben et al., 2021), maize (Carlson, 1978, 1986; Puertas et al., 2000; Blavet et al., 2021) and goat grass (Ruban et al., 2014, 2020) to understand the origin and evolution of Bs. Most studies have compared repetitive sequences in the As and Bs in these three plant species (Rimpau and Flavell, 1975; Stark et al., 1996) and demonstrated that Bs have the same sequence composition as that of A chromosomes.

The remarkable advances in NGS and ‘omics’ platforms have greatly enhanced our understanding of the origin of Bs and have provided significant insights into their evolution and confirmed the origin of Bs largely from A chromosomes (Martis et al., 2012; Ruban et al., 2017) with the addition of organelle DNAs (Ruban et al., 2014, 2020). In rye, the B sequences seemingly are derived from 3 to 7R autosomes with a significant representation of organellar genomes (Martis et al., 2012). A similar amalgamation of A paralogues and chloroplast and mitochondrial DNA sequences forms the Bs of the wild progenitor of bread wheat, the goat grass *A. speltoides* (Ruban et al., 2020). The study also pointed to the possibilities of DNA transfer from organelle to nucleus during Bs evolution. Blavet et al. (2021) suggested an ancient origin of maize Bs based on the observed homology between Bs sequences with their A counterparts. They for the first time obtained a quality B chromosome reference sequence in maize and identified 758 genic sequences in the 125.9 Mb sequence region of the B chromosome. The authors further conducted an in-depth comparative analysis of transposable elements (TEs) located on A and B chromosomes. Although a high similarity was found in the TEs, a significant variation was detected among the age of long terminal repeat (LTR) type of TE families, which suggested difference in the activation histories of both sets of chromosomes. The present repertoire of genes on maize B chromosome shows extensive evolutionary divergence than the sequences present on A chromosome and the genes detected on the B chromosome do not show synteny with a putative progenitor region on A chromosomes and rather show dispersed paralogs with the A chromosomes. Many copies of the gene sequences on the B chromosome exemplify a relaxed purifying selection (Blavet et al., 2021).

In contrast to the above account, a few reports, however, have documented subtle differences between As and Bs sequences. For instance, Appels et al. (1978) used radioactive *in situ* hybridization (ISH) to show the existence of differences in sequence organization between As and Bs in rye, for the first time. They isolated a highly repetitive sequence from A chromosomes and then re-localized them on the chromosomes using ISH and found that the A-chromosome-specific repetitive sequence could be localized only on the telomeric regions of A chromosomes and not in the Bs. Similar evidence demonstrating sequence differences between A and Bs came from the study of Tsujimoto and Niwa (1992) and Cuadrado and Jouve (1994). Cuadrado and Jouve (1994) used multiple sets of highly repetitive sequences from both wheat and rye and investigated their localizations on As and Bs by fluorescent ISH. The authors used three highly repetitive sequences from rye (pSc119.2, pSc74, and pSc34) and two ribosomal DNA clones from wheat (pTa71 and pTa794 for 25s-5.8s–18s and 5s rDNA, respectively). Out of five probes, only two probes, pSc119.2 and pSc74, hybridized to the telomeric regions of rye Bs while the remaining DNA clones did not hybridize to the Bs.

Dhar et al. (2002), based on their investigations on *P. lagopus*, provided evidence that massive amplification of 5S and 45S rDNA-like sequences results in the origin of the B chromosome. Recently, Kumke et al. (2016) validated the hypothesis of Dhar et al. (2002) based on NGS analysis in *P. lagopus* with and without Bs. The authors reported significant differences in the composition of As and Bs attributable to B-specific satellite repeats. These initial landmark studies laid the foundation of the current widely accepted theory that Bs originate from A chromosome complement by either chromosomal rearrangements, duplications of A chromosomes, or unbalanced segregation (Dhar et al., 2002; Valente et al., 2014; Rajičić et al., 2017).

In animals, a sex-chromosome origin of B’s has been reported as both X chromosome and Bs undergo a pycnotic cycle of condensation-decondensation during meiosis. Evidence from many animal species has now been documented to support the theory of origin of Bs from sex-chromosome (Perfectti and Werren, 2001; Camacho, 2005; Rubtsov et al., 2009; Camacho et al., 2011; Ventura et al., 2015; Utsunomia et al., 2016; Rajičić et al., 2017). Whole sex chromosome and a paracentromeric region of X chromosome and has been implicated in the origin of Bs in *E. plorans* (grasshopper) and the rodent group oryzomyine (López-León et al., 1994; Ventura et al., 2015; Pokorná and Reifová, 2021) (Figure 1). Interestingly, in Korean field mice (*Apodemus peninsulae*), it has been demonstrated that in populations from western Siberia, Bs originate predominantly from autosomes while they originate from sex chromosomes in populations from the Far East (Rubtsov et al., 2009), thus suggesting that populations of a species with different geographic origins may evolve Bs through different evolutionary pathways.

Various molecular cytogenetical studies in animals have provided experimental evidence for the interspecific origin of Bs by Perfectti and Werren (2001), who observed that centric fragments were generated from chromosome breakage during the interspecific transfer of chromosomes from *N. giraulti* into the jewel wasps *N. vitripennis*. The authors concluded that interspecific hybridization leading to unstable centric fragments can provide impetus to B chromosome generation. Further, the Bs sequence in jewel wasp was shown to resemble more with species of the genus *Trichomalopsis*, suggesting intergeneric hybridization (McAllister and Werren, 1997). In *Polycelis nigra*, which are pseudogamous parthenogenic flat worms, Bs may originate from the incompletely expelled As from the sperm (Sharbel et al., 1997).

A mono- vs*.* polyphyletic origin of B chromosome remains elusive. B chromosomes present in the cultivated *(S. cereale* ssp. *cereale*) and wild species (*S. cereale* ssp. *segetale*) of rye growing in diverse geographical locations share similar morphology and meiotic pairing behaviour indicating monophyletic origin of B chromosome in rye (Niwa and Sakamoto, 1996; Houben et al., 1999). Chromosome painting of seven B chromosomes in two fish species belonging to the genus *Astyanax* with 18S ribosomal DNA (rDNA), H1 histone genes and As51 satellite DNA (AC)_15_ microsatellite-based probes has shown that all Bs shared homologous DNA sequences not only amongst them but also with a variable number of A chromosomes in each species indicating a common origin for all seven Bs analysed (Duílio et al., 2016). The B chromosome in *Cestrum strigilatum* differs from the B chromosome of the other six species as it lacks the 5S rDNA sequence, which either might have been lost during B chromosome differentiation in this species or the B chromosome has evolved independently in this species (Vanzela et al., 2017).

## 5 Behaviour and transmission of Bs

The most well-understood explanation of the survival and transmission of Bs is through ‘drive’, which can occur in many ways during mitosis or at pre-meiotic, post-meiotic and also during meiotic divisions. Drive refers to a phenomenon in which the rates of transmission of chromosomes are higher than 0.5, either through female or male sex tracks, discounting Mendel’s law. This phenomenon was first observed in *Drosophila obscura* when some males gave rise to only female offspring (Gershenson, 1928). In the rye *S. cereale* and many other Triticeae species, a drive during the first pollen mitosis and/or first post-meiotic division is the most observed mechanism. Hakansson (1948, 1959), for example, observed anaphase cells with lagging Bs in the embryo sac during the first post-meiotic division in rye. Similarly, Mendelson and Zohary (1972) and Ohta (1996) observed a drive of Bs during first pollen mitosis in *A. mutica* and *A. speltoides*. Evidence has also accumulated to suggest that Bs themselves regulate the process of non-disjunction (Matthews and Jones, 1983; Romera et al., 1991). For example, on an introduction of the supernumerary chromosome of rye into the hexaploid wheat (Niwa et al., 1997; Endo et al., 2008), B non-disjunction occurs.

In maize, investigations have revealed non-disjunction of Bs at the second pollen grain mitosis (Carlson, 1969, 1978, 1986; González-Sanchez et al., 2003), due to which the B chromosome-containing sperm gets the benefit of fertilizing the egg. Additionally, it has been established by fluorescent ISH using the B-specific 157-bp ZmB satellite repeat sequence that Bs are positioned at the tip of sperm nuclei, which partly explains preferential fertilization by B chromosome-containing sperm (Shi et al., 1996; Rusche et al., 1997). Extensive studies have been undertaken in maize using A-B translocations and deletion lines to understand the role played by the centromere and different regions of Bs in controlling the non-disjunction process. It was discovered that the heterochromatic region 3 lying adjacent to the centromere (CenH3) is the sticking region for non-disjunction and a tiny fraction (approx. 700 kb domain) of the B-specific ZmB repeat sequence is needed to interact with the CenH3 (Jin et al., 2005).

Dhar et al. (2017) performed extensive crossing experiments in *Plantago lagopus* in order to study the mechanism of drive. The authors made crosses among 0B, 1B, and 2B plants and generated selfed progenies and cytologically analyzed them to determine the mode of inheritance of Bs. Fluorescent ISH and 5S rDNA probe was used in 1B and 2B plants to identify the B chromosome(s). The results explicitly showed that when 1B plant was used as a male parent, the transmission rate of Bs followed Mendelian laws, however, in the cases when it was used as a female parent, significant deviations from the 1:1 ratio were observed. These results thus point that in *P. lagopus* the preferential transmission of the Bs is through the female sex track. More recently, Silva et al. (2021) assessed the meiotic behavior of supernumerary Bs of *Psalidodon paranae,* a neotropical fish. They observed open self-pairing in the case of one B chromosome, whereas, in two Bs, separate close self-paired synaptonemal complexes were formed. Moreover, they also concluded that Bs show a self-pairing process to escape from meiotic silencing of unsynapsed chromatin, allowing expression of their own genes to facilitate formation of fertile individuals.

Despite the high susceptibility of Bs to mutations (Bakkali et al., 2003), only a few spontaneous B-A spontaneous translocations have been reported in *Z. mays*, *Narcissus*, *Lolium* and *Pennisetum glaucum* (Pushpa, 1980). Several B-A translocations in maize have been induced for genetic studies and more than 900 reciprocal translocations mostly between As and Bs have been recovered (Longley, 1952). Two interesting results have been derived from the analysis of B-A interchanges in maize (1) a reduced cross-over frequency in the region flanking the centromere (2) a cross-over frequency decrease in the vicinity of the breakpoints (Anderson et al., 1955). Kindiger et al. (1991) suggested that formation of a chain configuration between the A, A-B and B-A chromosomes is the consequence of the low frequency of chiasmata in the interstitial region of the A and B chromosomes facilitating increase in the probability of generation of gametes with A and A-Bs. Further, in rye, the induced B-A translocations are subjected to changes occurring during vegetative development (Hasterok et al., 2002). C-banding showed that in maize, A4 autosome and the BM8 chromosome are involved in translocation. The breakpoints for the translocation were located close to the interstitial C-band in the B and at the distal end of the A. The double FISH techniquefurther showed that the B chromosome gained an autosomal segment and lost an rDNA segment that was then transferred to A4 (Hasterok et al., 2002).

## 6 Perpetuation of B chromosomes

A unique non-disjunction of sister chromatids is proposed as one of the processes that regulates the Bs’ drive. There are marked differences in the As’ and Bs’ non-disjunction processes. It is well known that non-disjunction leads to genetic instability and cell death *via* the production of aneuploids in As, however, controlled non-disjunction favors Bs’ transmission by permitting their accumulation in the generative nucleus, as seen in the 2nd pollen grain mitosis in maize (Blavet et al., 2021; Chen et al., 2022). Further, non-disjunction results in one gamete containing duplicate Bs and the other lacking it, and a yet unknown support mechanism facilitates the preferential fertilization of gametes containing Bs thus ensuring their perpetuation. It is evident that non-disjunction of Bs occurs at an apparently higher level than As and has been attributed to functional differences in the centromeres, absence of lethal effect of gene-dosage or other mechanistic differences which still remain to be unravelled (Komluski et al., 2022). In maize B, a high-throughput genome sequence has revealed that a B-specific repeat sequence dispersed in and around the centromere acts as a cis factor for non-disjunction (Blavet et al., 2021). The presence of distinct centromeric and pericentromeric repeats like CentC repeats and specific retrotransposons disrupted with B-centromeric specific sequences in maize (Jin et al., 2005), ScC 111 a B-specific repeat and mtDNA (Banaei-Moghaddam et al., 2012) in rye differentiates them from standard As and play a significant role in non-disjunction. The failure in proper mitotic segregation during 1^st^ pollen mitosis in rye reflects the inability to precisely resolve heterochromatin in the pericentromeric region of Bs (Houben, 2017). Interestingly, B-mediated post-meiotic non-disjunction process in the 1^st^ pollen mitosis is observed to be autonomously regulated and works well when rye Bs are introduced into other species like *Triticum aestivum, Triticale* and *Secale vavilovii* as well (Jones, 2018).

Intriguingly, unlike A chromosomes, Bs remain stable as univalents probably through a distinct centromeric function (Birchler and Yang, 2021). The mechanism for this stability involves a B-specific centromeric repeat that shows precocious attachment to the spindle (González-Sánchez et al., 2007). In another non-conventional hack, the B chromosome somehow coaxes the cell division machinery to bring about crossing over between its heterochromatic structures. Increased recombination between self-paired Bs, especially in male meiosis in the heterochromatic regions assures their segregation and ultimately the transmission to the next generation (Chen et al., 2022). The existence and perpetuation of Bs is supported by various genic regions contained in them that control non-disjunction, and univalent stability (Birchler and Yang, 2021; Blavet et al., 2021; Boudichevskaia et al., 2022; Chen et al., 2022).

In fungi, the non-Mendelian segregation involves meiotic chromosomes drives and loss and duplication of chromosomes during meiosis (Orbach et al., 1996; Mehrabi et al., 2017; Fouché et al., 2018; Habig et al., 2018, Habig et al., 2021; Möller et al., 2018; Komluski et al., 2022). The inheritance of unpaired accessory chromosomes from only one of the parental strains to haploid meiotic progenies represents a transmission advantage process in fungi and was first reported for an accessory chromosome of *Cochliobolus heterostrophus* that causes southern corn leaf blight (Tzeng et al., 1992). Likewise, in the fungus *Leptosphaeria maculans*, as many as 83% of meiotic daughter progeny contained the mini-B chromosomes (Balesdent et al., 2013). The mechanistic details of transmission drives in these two species are not well understood, though it is speculated that the transmission advantage might involve some adaptive significance conferred by the presence of Bs (Komluski et al., 2022). A meiotic drive mechanism has been revealed in the wheat pathogen *Z. tritici* that affects only the unpaired Bs inherited from the female parent (Habig et al., 2018). Interestingly, the inheritance of the same unpaired Bs from the male parent of the same strain showed strict Mendelian transmission. Further, Mendelian segregation and recombination was observed in the Bs that had a homolog in both the parental strains (Habig et al., 2018). The observed meiotic drive, therefore, has been proposed to be supported by a selective replication/amplification of unpaired Bs from the female parent and preferential elimination of all paired Bs (Komluski et al., 2022).

Further, the fungal accessory chromosomes get frequently removed during mitosis or meiosis with a differential rate of mitotic loss as exemplified by *Fusarium oxysporum f*. sp. *lycoprsici* (1 in every 35,000 spores) and *Z. ardabiliae* (1 in every 50 spores) (Vlaardingerbroek et al., 2016; Habig et al., 2021). Interestingly, histone H3K27me3-induced aberrant DNA replication that destabilizes the Bs by increasing their mutation rates has been proposed to be responsible for such high B chromosome losses. It is important to note that H3K27me3 is restricted to sub-telomeric regions on As, while being present on the entire length of Bs. The entire B chromosome thus might get associated to the nuclear envelope affecting the DNA replication and transmission process during mitosis. A similar loss of chromosomes and segregation distortions have been observed during fungal meiotic drives as well. A 5% loss of conditionally dispensable chromosomes (CDC) of *Leptosphaeria maculans* has been recorded (Balesdent et al., 2013). Non-disjunction of homologs at Meiosis I or II has been held responsible for loss of supernumerary chromosomes in meiotic progenies of rice blast fungus *M. oryzae*, *Z. tritici* and *N. hematococca* MPVI (Orbach et al., 1996; Fouché et al., 2018; Habig et al., 2018). It is fundamentally important to understand the mechanism of differential differentiation achieved between the As and Bs. The recent reports of differential histone epigenetic marks in the form of H3K27me3 distribution (Habig et al., 2021) in fungal As and Bs hint at the involvement of subtle epigenetic regulatory mechanisms and warrant detailed future B-related epigenetic studies across eukaryotes.

In animals, the drive occurs during meiosis in contrast to post-meiotic drives in plants. The female meiotic drive has been extensively studied in grasshoppers and the mealybug *P. obscurus* and represents the most common mode of Bs transmission. The preferential segregation of Bs univalent is postulated as one of the mechanisms of Bs drive (Hewitt, 1976; Nur, 1977; Jones, 2017). At the population level in both plants and animals, B equilibrium frequency differs among populations. Interestingly, while Bs provide the drive, the standard As attempt to limit the increase in the number of Bs (Houben, 2017; Jones, 2018). The grasshopper *Eyprepocnemis plorans* represents an interesting case where suppressor A genes neutralize one of the B types culminating in its elimination from the population. Nevertheless, a neo B may arise again with an active drive mechanism. Similar evidence of A-suppressed B drives exists in maize and rye, though there are no instances where As have completely suppressed and neutralized any Bs (Houben, 2017; Jones, 2018).

Although the above observations have been made in taxa that possess unusual drive mechanisms, in general, these selfish entities secure their perpetuation in the populations and seem to have devised some or the other non-conventional endurance mechanisms including even conferring advantages to the hosts. With the usage of molecular cytogenetics and genome sequencing techniques, several phenotypic and regulatory functions such as seed germination, antibiotic resistance and pathogenicity, fitness, stress tolerance, sex determination, and transcriptional regulation of genes located on A chromosome (Yoshida et al., 2011; Banaei-Moghaddam et al., 2013; Lin et al., 2014; Huang et al., 2016; Habig et al., 2017; Pereira et al., 2017; Park et al., 2019; Blavet et al., 2021; Ma et al., 2021; Boudichevskaia et al., 2022; Shi et al., 2022) have been assigned to Bs. Bs have even been observed to turn into sex or germline-restricted chromosomes making them an essential part of the genome, thereby, contributing to organisms’ fertility (Pokorná and Reifová, 2021).

In short, Bs show immense dynamism in their perpetuation and survival strategies that are manifested in the form of diverse drives and non-disjunction mechanisms, conferring advantages to their hosts besides getting translocated to sex chromosomes or autosomes or acquiring stable segregation, inheritance and maintenance in the populations by initiating pairing between two B chromosomes (Camacho et al., 2000; Houben et al., 2000; Cabrero et al., 2003; Hsu et al., 2003). Their presence in populations most likely seems to be determined as a trade-off between the drive efficiency and adaptive significance versus the adverse effects on reproduction. In low numbers, Bs have no significant effect, however, they may have a negative impact on fertility and fitness of the organism, when present in high numbers (Habig et al., 2017; Pereira et al., 2017; Chen et al., 2022).

## 7 Genetic basis of Bs’ transmission

The research on molecular analysis of Bs has shown exponential growth in recent years. NGS data, which has largely been accumulated in species that possess drive, has provided information on both protein-coding genes and repetitive DNA contained in Bs. The plant species *S. cereale* (rye), *Z. mays* (maize) and *A. speltoides* (goat grass) and the animal species *E. plorans* (grass hopper), *N. vitripennis* (jewel wasp), *L. migratoria* (locust), and *Astyanax mexicanus* (cavefish) represent such examples wherein, interesting incidents on the genetic control of drive process have been documented (Camacho, 2022).

In rye, Lima-de-Faria (1962) indicated that the heterochromatin block present in the long arm of rye B contains a controlling element for Bs drive. This observation was supported by Puertas et al. (1998). Two satDNA repeat families E3900 and D1100 that are specific to B (Langdon et al., 2000) transcribing to produce heterogeneous non-coding RNAs were then found to be associated with this control mechanism. The non-disjunction of rye Bs that arises due to the cohesion of sister chromatids of Bs and the asymmetric spindle formation at the first pollen mitosis results in Bs accumulating in the generative nucleus (Camacho, 2022). No genes hitherto have been demarcated to be controlling the rye Bs drive but the discovery of several protein-coding genes by Martis et al. (2012) engenders future research for the identification of genes controlling the drive.

Species-specific B-centromeric sequences in maize disrupt the CentC repeats and centromere-specific retrotransposons on the Bs (Jin et al., 2005). Such B-specific sequences at the centromere are essentially involved in creating an asymmetric distribution of the chromosomes contributing to the ‘drive mechanisms’ (Yamagishi et al., 2008). Blavet et al. (2021) with NGS sequencing have recently indicated that 758 B-located putative genic sequences might be correlated with functions like non-disjunction, preferential fertilization, and stabilization of univalent, the factors that contribute to the drive mediated Bs transmission in maize.

In goatgrass, the Bs are limited to aerial parts and are conspicuously eliminated in the roots. Ruban et al. (2020) recently elaborated the Bs elimination process in roots as part of a controlled process wherein chromatid lagging and non-disjunction during the mitotic anaphase leads to generation of B-containing micronuclei that are eventually degraded to eliminate Bs from the roots. Further, by NGS, the authors found 229 genes presumed to be located on the Bs. Future research is anticipated to explore the involvement of some of the B genes in the above processes to support Bs transmission.

The molecular underpinnings of the non-mendelian drive mechanisms of Bs in animals are limited as their NGS data has been accumulated only in a few species like *N. vitripennis, D. melanogaster*, and *A. mexicanus*. In the fruit fly*,* mitotically unstable Bs, without an apparent drive have been reported (Bauerly et al., 2014). Interestingly, high throughput sequencing of fruitfly Bs has revealed several highly repeated elements, while the protein-coding genes have been reported to be conspicuously absent in the fruitfly Bs. The mitotic instability of these Bs has rather been associated with a process known as gonotaxis, which is defined as the tendency of Bs to move preferably to the germline during mitosis or meiosis (Burt and Trivers, 2006).

Palestis et al. (2004, 2010) provided evidence in support of the hypothesis of “centromeric drive” in both mammals and orthopteran insects. According to this hypothesis, in female meiosis, chromosomes that can make more microtubule attachments by having a centromere are preferred as compared to those which have fewer microtubular attachments. The centromeric region along with telomeres represent the primary heterochromatic regions of the chromosome.

High quality NGS-mediated genome sequencing and assembly has identified 44 and 63 genes in the Bs of *N. vitripennis* and *A. mexicanus*, respectively (Dalla Benetta et al., 2020; Imarazene et al., 2021). The best evidence to link Bs genetic system to the transmission drive mechanism is the jewel wasp gene *haplodizer*, which is a paternal sex ratio (PSR) linked gene. Likewise, in the cavefish, *growth differentiation factor 6b (gd6b)* a master sex-determining gene has been located on the B chromosome (Imarazene et al., 2021). However, the presence of high sequence similarity between the paralogous copies of the As and Bs holds back assigning the exact role of sex determination in the species to Bs. Further research is required to develop clarity on the above aspects. Besides the above three species, *E. plorans* (grasshopper) and the locust *L. migratoria* have revealed ten and 25 protein-coding genes, respectively on their Bs (Navarro-Domínguez et al., 2019; Ruiz-Ruano et al., 2019). While the grasshopper B-genes code for cell division, one of the locust B-gene *apc1* has been shown to code for an *E3 ubiquitin ligase* gene that mediates metaphase-anaphase transition during the cell division and also for the large subunit of the anaphase-promoting complex (APC) also known as cyclosome (Ruiz-Ruano et al., 2019; Camacho, 2022). The possible role of higher APC1 protein in B-containing cells in pushing both the B chromatids to one pole during metaphase-anaphase transition is an interesting future research domain.

Interestingly, sequencing of fungal B chromosomes has revealed distinct genomic properties like more gene duplications, increased density of TEs and higher evolutionary rates than the As (Ma et al., 2010; Grandaubert et al., 2015). A faster-evolving B has been suggested to benefit pathogens by permitting frequent B mutations resulting in rapid transmission and adaptations to host in the new environments (Yang et al., 2020).

As clear from the above account, although efforts have been done for genome sequencing and assemblies on the NGS platforms to understand and unravel the mechanisms associated with the drive and transmission of Bs in plants, fungi and animals, a lot needs to be done for assigning meaning to the sequenced data. A clear-cut identification of putative B-genes and allocating them functions will probably take more time.

## 8 Bs associated adaptive advantages to the host

For a long time, Bs were considered genetically inert elements, utilizing replication machinery of the host like a parasite for their survival through drive (Murray, 1984; Jones, 1991). They were thought to provide no advantage to their hosts and rather produced negative phenotypic effects and reduced fertility when present in high copy numbers (Bougourd and Jones 1997; Camacho et al., 2004). For instance, in rye, the existence of Bs under normal growth conditions impart a reduction in height, weight, seed weight, and tiller numbers almost in proportion to the number of Bs contained (Müntzing, 1963; Moss, 1966). In *S. purpureosericeum*, the Bs-associated phenotypic effects emerged as smaller plants with reduced fertility and a lower number of seeds. It was interesting to note that the intensity of the effect directly correlated not with the number of Bs but with the amount of Bs DNA in the nucleus (Karafiátová et al., 2021). Similarly, previous observations in rye and maize emphasized that adverse effects of Bs on plant fitness get pronounced when the B DNA mass exceeds 20% of the total mass of DNA present in the nucleus (Bartoš et al., 2008; Jones et al., 2008; Martis et al., 2012; Karafiátová et al., 2021). So, the genome content of 2 Bs in *S. purpureosriceum* is comparable to 10 Bs in maize and 6 Bs in rye in producing an equivalent adverse effect on plant fitness and fertility. In this regard, since bigger chromosome size and the larger B’s genome content would lead to sterility and eliminate Bs from the populations, the trend of reduction in size would be favored by B’s evolutionary dynamics to preserve them in the populations (Karafiátová et al., 2021).

In contrast to the above few reports, however, recent evidence showcases the advantages offered by Bs to the hosts, especially in species that do not possess a drive mechanism. This feature is considered as yet another evolutionary adaptation route taken by Bs to maintain their survival in diverse plant, fungal and animal species (Müntzing, 1954; Jackson and Newmark, 1960; Moss, 1966; Williams, 1970; Dover and Riley, 1972; Dherawattana and Sadanaga, 1973; Rees and Hutchinson, 1974; Puertas et al., 1987; Staub, 1987; Holmes and Bougourd, 1989; Miao et al., 1991; Plowman and Bougourd, 1994; Enkerli et al., 1997; Burt and Trivers, 1998; Hatta et al., 2002; Nokkala et al., 2003; Rodriguez-Carres et al., 2008; Akagi et al., 2009; Coleman et al., 2009; Ma et al., 2010; Yoshida et al., 2011; Kousaka and Endo, 2012; Balesdent et al., 2013; Houben et al., 2014; Thatcher et al., 2016; Williams et al., 2016; Dalíková et al., 2017; Pereira et al., 2017; van Dam et al., 2017; Ruban et al., 2017; Armitage et al., 2018; Plaumann et al., 2018; Kinsella et al., 2019; Torgasheva et al., 2019; Ahmad et al., 2020; Dalla Benetta et al., 2020; Birchler and Yang, 2021; Imarazene et al., 2021; Lewis et al., 2021; Pokorná and Reifova, 2021; Boudichevskaia et al., 2022; Camacho, 2022; Chen et al., 2022; Jonika et al., 2022; Komluski et al., 2022; Shi et al., 2022).

For example, in *Allium schoenoprasum* (chive plant), and ryegrass (*Lolium perenne* L.), individuals with Bs displayed better survival rates than those devoid of them in their natural habitats (Rees and Hutchinson, 1974; Holmes and Bougourd, 1989; Miao et al., 1991; Plowman and Bougourd, 1994). Likewise, the rye Bs were shown to mitigate heat stress for the host plants (Pereira et al., 2017). An in-depth cytogenetic and molecular analysis was conducted in rye plants carrying 0 and 2Bs to understand the expression of the *Hsp101* gene and the E3900 and tE3900 (truncated) under heat stress during meiotic stages and various tissues. The heat-stressed anthers from 2B plants showed a marked increase in the level of E3900. Most importantly, a significant up-regulation of the truncated version of E3900 (tE3900) was observed in anthers at pachytene under heat-stress, which was about three-fold in 0B plants and about 40-four-fold upregulation in 2B plants. Collectively, the results suggest rye Bs implications for heat tolerance. Notably, in the rye, both beneficial function and drive have been reported and their co-occurrence during the evolution of Bs points towards a much more complex nature of rye Bs (Pereira et al., 2017).

**FIGURE 2:**
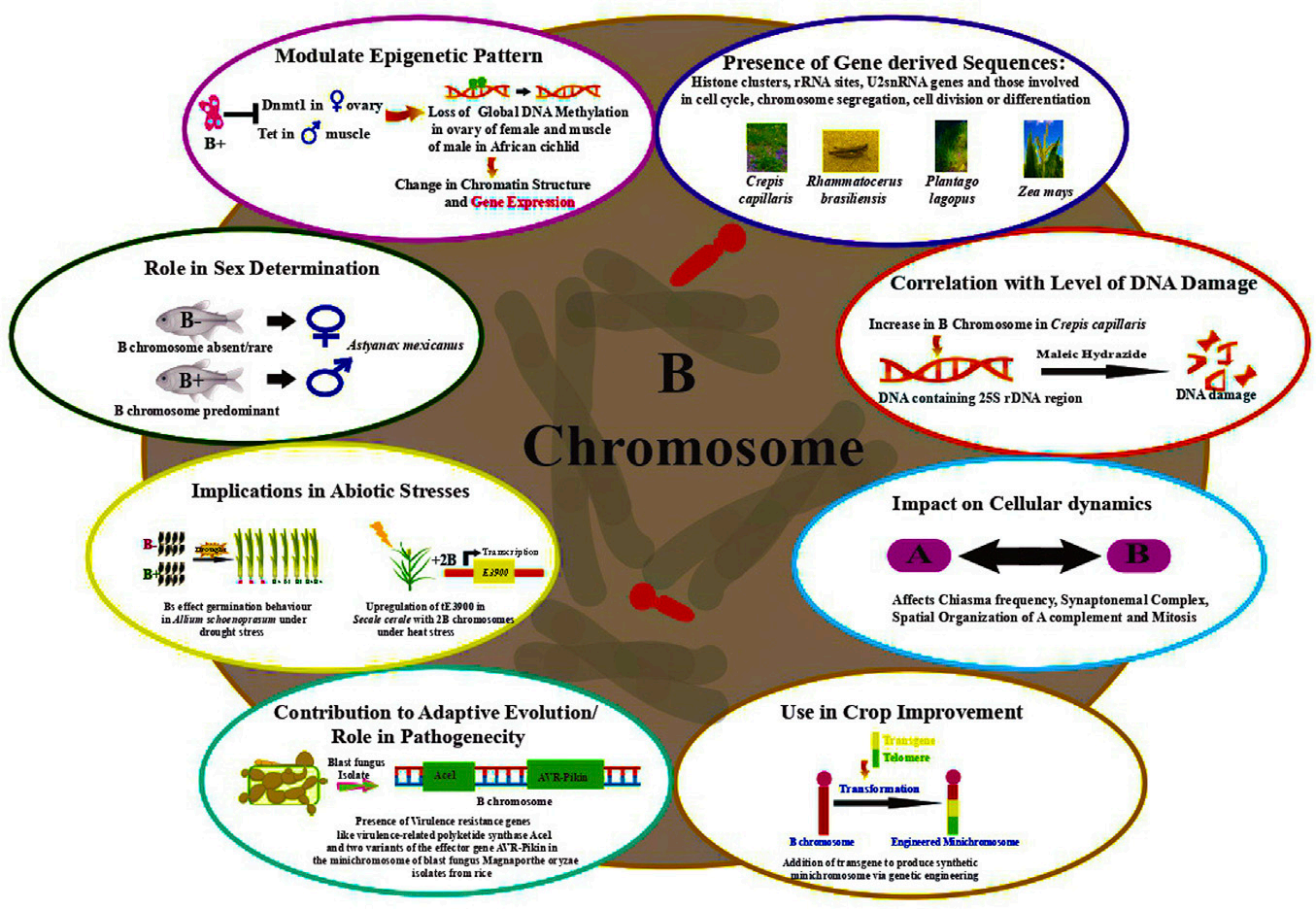
Functional aspects of genes identified on B chromosomes in various organisms.

Dherawattana and Sadanaga (1973) observed that Bs impart resistance to rust caused by *Puccinia coronata* f. sp. *avenae* in *Avena sativa*. Many studies have ascribed an adaptive role to Bs in terms of imparting phenotypic advantages (Jones, 1991), influencing the distribution of chiasma and hence the rate of recombination in populations (Rees, 1974). Interesting instances of the adaptive significance of Bs presence have been documented in the Eastern Siberian spring variety wherein grains germinating at low temperature showed fewer Bs (Moss, 1966). A direct correlation was drawn between the increase in seed weight and the coefficient of variation in the number of grains in the species bearing B chromosome. Additionally, the cumulative effect of Bs, depending on their total number in a cell has been reported on phenotypes including achene color in *Haplopappus gracilis* (Jackson and Newmark, 1960), meiotic pairing in *A. mutica* (Dover and Riley, 1972), hybrids between common wheat and *Ae. variabilis* (Kousaka and Endo, 2012), and leaf striping in maize (Staub, 1987). Figure 2 depicts the functional roles ascribed to B chromosomes in various organisms.

Many reports in the past decade have confirmed the beneficial roles played by Bs in a series of fungal species. Presence of Bs impart resistance in the fungus *N. haematococca* to antibiotics which are naturally produced by pea plants (Enkerli et al., 1997). Bs-associated increase in pathogenicity has been demonstrated in many other fungi including *M. oryzae* (Balesdent et al., 2013; van Dam et al., 2017), *F. oxysporum* (Balesdent et al., 2013; van Dam et al., 2017), *F.* sp. *radicis-cucumerinum* (Balesdent et al., 2013; van Dam et al., 2017), *Alternaria alternata* (Balesdent et al., 2013; van Dam et al., 2017), *Leptosphaeria maculans and C. heterostrophus* (Ma et al., 2010; Balesdent et al., 2013; van Dam et al., 2017). The latter authors sequenced accessory chromosomes of *F. oxysporum* and showed that a central part of this chromosome contains homologs of *SIX6*, *SIX9*, *SIX11*, and *SIX13* genes, the potential candidate effector genes. Of these, the role of *SIX 6* in imparting virulence to the pathogen has been demonstrated by studying mutants in which the *SIX6* locus was disrupted. A clear reduction in virulence was detected in mutants having disrupted the *SIX6* locus. Similarly, Plaumann et al. (2018) investigated the role of Bs in the hemi biotrophic plant pathogen *Colletotrichum higginsianum,* which infects species of the family *Brassicaceae*. This pathogen has two dispensable chromosomes, chromosomes 11 and 12, which share genome sequences between them but differ from the core genome. Chromosomes 11 and 12 are much smaller in comparison to the other 10 chromosomes, show a lower gene density, and are rich in transposable elements and genes encoding potential effector proteins. Chromosome 11 specifically plays an important role in conferring pathogenicity to the fungus by suppressing post-invasion plant defense mechanisms.

Further, the existence of Bs has also been directly correlated with the breeding systems in plants. Burt and Trivers (1998) performed a comparative study among diverse species of British flowering plants and reported that Bs are present frequently and in larger numbers in outbreeding species than in inbreeding species. Many experimental studies have also emphasized the significant role of the breeding system in the evolution of Bs. Müntzing (1954) reported that B chromosome frequency decreased when outbreeding rye was inbred. Similarly, Puertas et al. (1987) showed that the experimental introduction of Bs in the inbreeding species of rye, *S. vavilovii*, declines the number of Bs rapidly. The presence of more Bs in outbreeding, and cross-pollinating populations than the inbreeding self-pollinating populations indicates some adaptive role assigned to the Bs in evolutionary advancement.

In animals, Bs imparting adaptive significance have been reported in the frog *Leiopelma hochstetteri* (Yoshida et al., 2011), moths *Tischeria ekebladella* (Dalíková et al., 2017), *Plutella xylostella* (Dalíková et al., 2017), *Iulia ohridella* (Dalíková et al., 2017), and iulia butterfly *Dryas iulia* (Lewis et al., 2021), passerie birds (Kinsella et al., 2019; Torgasheva et al., 2019) and fish *A. mexicanus* (Imarazene et al., 2021). It is interesting to note that Bs might behave as sex chromosomes themselves as described in cichlid fish and are also proposed to participate in sex determination (Camacho, 2005; Clark and Kocher, 2019; Jonika et al., 2022). Sex chromosome system lability is a well-known phenomenon, and the possibility of losing an existing sex chromosome and incorporation of new genomic regions and/or involvement of B chromosomes is suggested as a possible canonical origin of sex chromosomes (Jonika et al., 2022). Bs and Y/W are gene-poor chromosomes and by mechanisms like transposition of ancestral Y-determiner from Y or emergence of a novel determiner (Pan et al., 2021), Bs can acquire a sex-determining factor. Literature shows examples of sex transition in Bs where in conditions like XO and ZO, they might mimic Y and W and pair with X and Z, chromosomes, respectively (Pokorná and Reifova, 2021). Recruitment of Bs for sex determination are exemplified by instances in Lepidopteran W chromosome corresponding to a B chromosome gaining a factor for femaleness (Fraïsse et al., 2017) and W representing a captured B in *D. iulia (* Lewis et al., 2021). The recent genome sequencing in butterfly *D. iulia* supports the origin of W chromosomes from Bs and suggests the multiple occurrences of this event during their evolution (Lewis et al., 2021). Various canonical models like sex chromosome turnover, Z-autosome fusion, and the non-canonical models like B chromosome fusion have been hypothesized for the origin of the giant sex chromosome of cichlid fish (Conteet al., 2021; Lewis et al., 2021).

In cavefish *A. mexicanus* and *A. scrabipinnis*, Bs behave like a male determining univalent Y with males having many copies of the same Bs; and exists as a macro B (possibly W) found in 30% of the females and none in the males, respectively (Mizoguchi and Martins-Santos, 1997; Imarazene et al., 2021). Further, B-influenced sex determination in cichlid *Lithochromis rubripinnis* is more frequent in females which also shows a B-dosage effect with mother females carrying single copy of B producing at least 70% female clutches, while 100% female clutches were observed if two copies of Bs were present in the mother cichlids (Yoshida et al., 2011).

Evidence has also been put forth to suggest the origin of the Y chromosome from the Bs in fruit fly *D. melanogaster*, insect *Cacopsylla peregrina,* and cichlid fish of the tribe Oreochromini (Conte et al., 2021; Jonika et al., 2022). Y is a genic poor chromosome and none of the Y-linked genes find a homolog on X, and since all the identified paralogs lie on autosomes it was proposed that the Y chromosome might be B-derived in the fruit fly (Carvalho, 2002) or may be a product of fusion of ancestral Y with an autosome (Bachtrog, 2013).

Bs’ adaptive potential has further been highlighted in the characid fish *A. scabripinnis,* where Bs frequency has been correlated with the variation in altitudes and higher stretch along the same stream in three populations (Porto-Foresti et al., 1997; Néo et al., 2000). Macro Bs were present in two high altitudinal (1800 m and 1920 m) populations and were conspicuously absent in the low (700 m) altitude population. The observations support the parasitic theory of Bs evolution and propound that Bs thrive in populations growing in favorable environmental conditions and provide a selective advantage on fish in the higher stretches or altitudes.

## 9 Assigning functions to Bs: Identification of genes on B chromosomes

**FIGURE 3:**
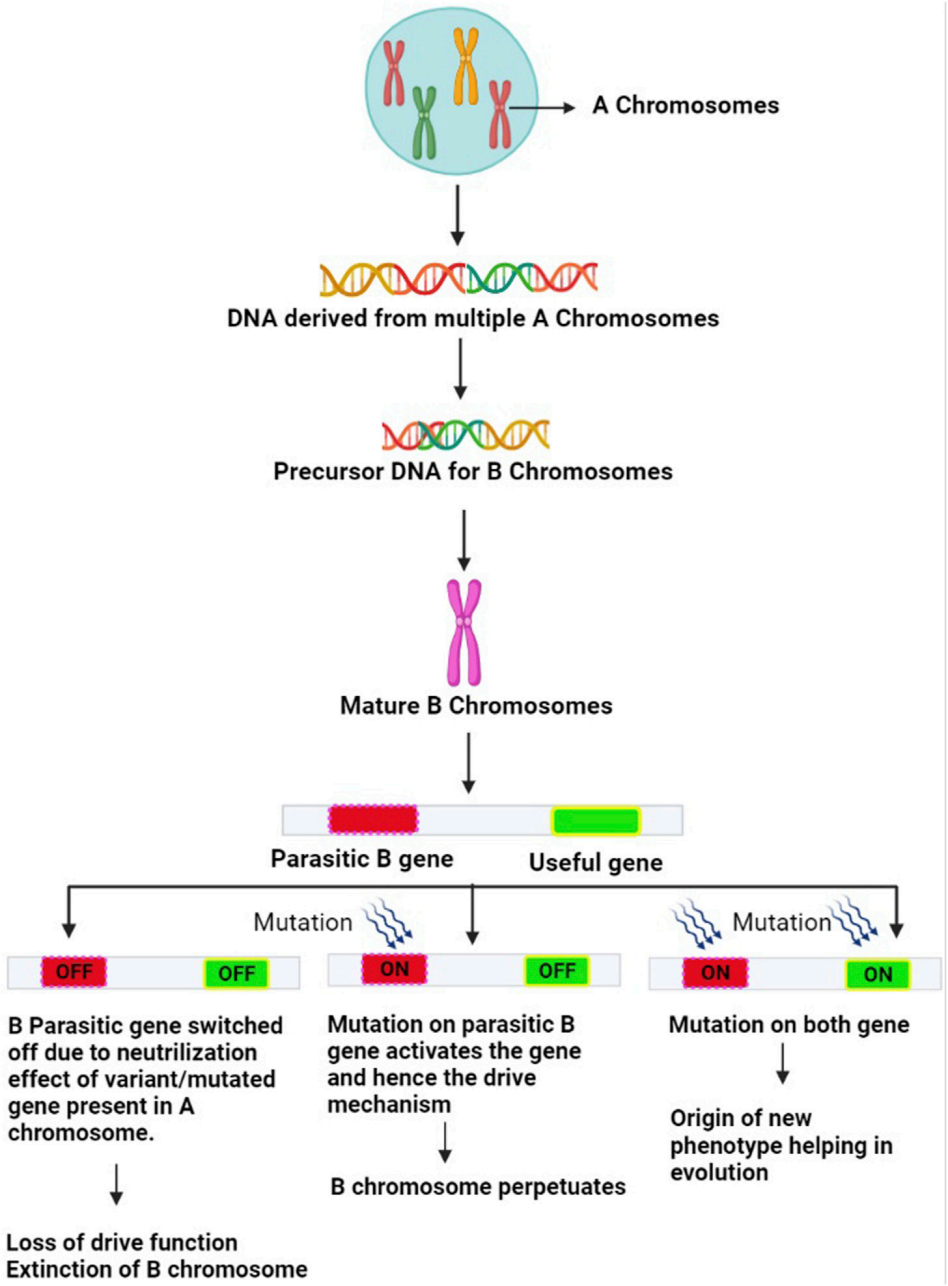
Mechanism of evolution of B chromosomes: The A chromosome-derived Bs acquire diverse types of DNA sequences. Certain variant/mutated genes of the A genome suppress the parasitic gene on B and neutralize their harmful effects. The B-parasitic gene, however, is essential for its drive. There may be three outcomes of this suppression mechanism. (1) Genes on B chromosomes do not undergo any mutation, and remain switched off eventually resulting in loss of B drive furthering their extinction (2) Parasitic gene on B chromosome undergoes mutation and gets switched on to overcome the neutralizing effects of the A variant genes. This revives the B drive mechanism leading to the regeneration of B chromosomes (3) Both the parasitic and useful genes on B chromosome undergo mutations and get switched on. As a result, the drive mechanism becomes active and Bs are regenerated and the useful gene expresses itself leading to a new phenotype that provides adaptive evolutionary advantage to the host harboring this useful mutated gene.

A plethora of investigations conducted in the past decade across organisms using cutting-edge next-generation sequencing, genomics and transcriptomics technologies coupled with traditional and molecular cytogenetics tools (Houben et al., 2014, 2021; Ma et al., 2017; Ruban et al., 2017; Valente et al., 2017; Dhar et al., 2019; Dalla Bennetta et al., 2020; Chen et al., 2022; Shi et al., 2022) have shown the presence of non-coding transcribed sequences, protein-coding genes, pseudogenes, multigene families such as H1, H3 and H4 histones, transposable elements and repetitive sequences on Bs. The majority of these genes are involved in regulating cell division or differentiation and cell cycle, kinetochore structure, microtubule organization, the transition from metaphase to anaphase, chromosome segregation, recombination, chromosome non-disjunction and also influence the expression of genes on A chromosomes (Valente et al., 2014; Navarro-Domínguez et al., 2017; Makunin et al., 2018; Marques et al., 2018; Ruiz-Ruano et al., 2019; Martins and Jehangir, 2021). A whole lot of these reports have been compiled in the form of many reviews (Croll and McDonald, 2012; Makunin et al., 2014; Banaei-Moghaddam et al., 2015; D’Ambrosio et al., 2017; Marques et al., 2018; Dalla Benetta et al., 2019; Ahmad and Martins, 2019; Ahmad et al., 2020; Jones and Ruban, 2019; Park et al., 2019; Houben et al., 2019; Ahmad and Martins, 2019). The various techniques used in Bs research are listed in Table 1. With the overwhelming evidence accumulated using the technological advancement, the long-standing interpretation of Bs as non-functional players now stands corrected. It is now well understood that Bs can regulate both direct and indirect changes across the whole transcriptional profile of the cell and therefore impose equal scrutiny. The presence of functional genes has allowed Bs to maintain their survival (Figure 3). In the present review, we have collated the information published on the identification of functional genes on B chromosomes between the years 2020–2022 in Table 2.

**TABLE 1:** Techniques used in the studies related to B chromosome research.

Technique	Details/Application	Reference
Mitotic and Meiotic analysis	To distinguish B chromosomes on the basis of their number and morphological aspects of size, centromere position and length of long (p) and short (q) arms and to determine meiotic behavior of B chromosomes with respect to chiasma and recombination frequency and anaphase segregation behavior	Thomson et al. (1984)
FISH (Fluorescence *in situ* Hybridization)	Physical mapping of highly repetitive and gene sequences using isotopic and non-isotopically labeled probes on *in vivo* mitotic metaphase preparations	Wilkes et al. (1995)
Chromosome Microdissection	Used to physically separate Bs or specific B-related sequences based on the use of a glass needle or laser capture-based chromosome dissection	Houben et al. (2001)
AFLP (Amplified Fragment Length Polymorphism)	Based on selective amplification of a selective subset of restriction fragments of genomes, AFLP finds uses in comparative analysis of genomes with and without B chromosomes in a species. Analysis has further been utilized to isolate B chromosome-specific DNA sequences	Qi et al. (2002)
Flow Sorting	Used to sort out B chromosomes or specific sequences from B chromosomes based on differences in light scattering and fluorescence parameters and indirect estimate of their size	Kubaláková et al. (2003)
GISH (Genomic *In situ* Hybridization)	To determine the homology of DNA sequences within B chromosomes and also with A chromosomes at intra and interspecific levels using the total DNA as genomic probes in an *in situ* hybridization experiment	Xie et al. (2014)
qPCR (Quantitative Polymearse Chain Reaction)	A robust quantitative method, amenable to automation, and is used to determine exact relative or absolute quantities of specific amplified sequences on B chromosomes	Fantinatti and Martins, (2016)
NGS (Next-Generation Sequencing)	A parallel ultra high-throughput, fast and scalable sequencing technology that is used to understand genomic composition, gene sequences, structural rearrangements and gene density	Fantinatti and Martins, 2016; Ruban et al., 2017
GO (Gene Ontology) term enrichment analysis	It is used for interpreting sets of genes by assigning them to a set of predefined bins depending on their functional characteristics	Ahmad and Martins, (2019)
WGBS (Whole Genome Bisulfite Sequencing)	The technique is used for methylome studies related to genome-wide DNA methylation changes at the single-nucleotide level. Useful for assessing B-linked epigenetic changes in the genome, and was first used for epigenomic profiling of the B chromosome *Aegilops* species	Ahmad et al. (2020)
ViSEAGO package of R (Bioconductor)	Used for functional annotations and gene ontologies enrichment analysis. ViSEAGO has been used for functional genomics analysis of B chromosome genes against the reference genes. The latest version of gene ontologies (GO) databases are loaded in R for each species from Ensembl and functional enrichment analysis is performed	Ahmad et al. (2020)
RNA-seq	RNA-seq analysis uses high-throughput sequencing to study transcriptomes of a cell with extreme refinement to provide higher coverage and better resolution as compared to previous micro-array-based methods and Sanger’s sequencing. Comparative RNA-seq analysis with and without B’s is an important tool to study the differential expression of A derived transcripts in the entire genome	Boudichevskaia et al. (2022)

**TABLE 2:** Functional characterization of B-linked genes in plants and animals^a^.

Organism	Inference	Reference
*Aegilops speltoides*	Transcriptomic analysis of *A. speltoides* embryos with and without Bs was employed and 341 B-unique transcript isoforms were identified. Of these, 70 were functionally annotated. In addition to development and signaling, genes regulating kinetochore function, spindle checkpoint and chromosome segregation were suggested to govern the selective elimination of Bs in roots of *A. speltoides*	Boudichevskaia et al. (2020)
*A. speltoides*	Chromosome elimination in the roots of *A*. *speltoides* was analyzed. Elimination of Bs was indicated to aid root tissues to survive tide-over accumulation of detrimental B-root specific transcripts. Twenty B-specific genic regions showed root-specific expression	Ruban et al. (2020)
*Secale cereale*	Comparative proteomics showed that Bs influence the proteome and biological functions of the host. 31 out of the 319 features were observed to be B-linked peptide features. The identification of a B-specific protein fragment with similarity to a glycine-rich RNA binding protein, however, showed differences from its A counterpart by two amino acids	Ma et al. (2021)
*S. cereale*	Comparative RNA-seq analyses in anthers of rye and wheat with and without additional rye Bs were conducted and B chromosome responsive A-located genes were found in both rye and wheat with + 2B and + B, respectively. Bs were found to influence A chromosome encoded processes such as “chromosome organization”, “chromatin silencing”, “DNA methylation”, and “gene silencing”. In addition, genes like Nuf2 involved in cell division-related functions indicate their relevance in maintaining Bs	Boudichevskaia et al. (2022)
*Zea mays*	Comparative transcriptomics led to the identification of 32 novel B-specific transcripts. Of these, 20 were confirmed to be B-specific. Bs in maize were found to harbor transcriptionally active sequences throughout the chromosomes and have been suggested to affect the expression of genes from A complement	Hong et al. (2020)
*Z. mays*	NGS sequencing platform was employed to discover the differential expression of 18 miRNAs in all tissue types such as pollen grains, leaves and roots. The targets for these miRNAs were identified as transcription factors. Three miRNAs were mapped to Bs and suggested this can affect the expression of A-derived transcription factors and miRNAs that in turn affect the expression of A-derived genes	Huang et al. (2020)
*Z. mays*	NGS methodology was employed to sequence, annotate and analyze Bs from maize. 758 protein-coding genes were identified from 125.9 Mb of B chromosome. Of these, transcript accumulation of at least 88 was found. In addition, a distant origin of Bs from A chromosomes was also suggested	Blavet et al. (2021)
*Z. mays*	Bs presence resulted in transcriptional and posttranscriptional changes in the gene expression of both A and B-linked genes. There were Cumulative and non-cumulative effects observed during the expression of genes, TEs and miRNAs. As many as 253 B-located genes were actively expressed in the leaf tissue. Differential expression of A- located genes was observed by the mere presence of B-genes, while a dosage effect depending upon the copy number of Bs was correlated to the expression of B-linked genes	Shi et al. (2022)
*Astyanax mexicanus, A. correntinus and A. flavolineata*	Genome sequencing led to the identification of B-specific sequences and genomic rearrangements in Bs. Functional annotation of B sequences revealed euchromatic regions on the Bs with novel, fragmented, and intact genes, regulating important biological processes. The findings suggest that the acquired DNA sequences favoured the drive	Ahmad et al. (2020)
*Characidium gomesi*	Fifty-nine satellite DNA families were found constituting the satellitome of the species, six SAT DNAs showed hybridization with the Bs, and showed specificity to sex chromosomes, autosomes, and Bs. The results suggested sex chromosomes origin for Bs. The hybridization of five other repeat families revealed the homology of Bs DNA with *C. gomesi* than other *Characidium* species. Bs were suggested to have the intraspecific origin in *C. gomesi* and sex chromosomes were indicated to be ancestors for Bs	Serrano-Freitas et al. (2020)
*Nasonia vitripennis*	NGS platform was employed to analyze the sequence composition and associated expression of paternal sex ratio (PSR), a B chromosome in the jewel wasp. PSR is responsible for the conversion of females to males and destroys the sperm’s hereditary material in young embryos to drive. A PSR-linked gene*, “haploidizer”* that specifically expresses in testis, was functionally validated by RNA interference (RNAi) to facilitate genome elimination and sex conversion	Dalla Banetta et al. (2020)
*A. mexicanus*	The putative master sex-determining gene gdf6b occupied two distinct loci at the Pachón male-predominant B chromosomes. Two duplicated copies of the growth differentiation factor (gd6b) were functionally characterized Bs from Pachón cavefish. Accumulation of gdf6b transcripts was found in differentiating male gonads and its knock-out induced sex reversal. Pachón B is termed a B-sex chromosome, by virtue of its functions as a putative male-sex-determining gene	Imarazene et al. (2021)
*Astatotilapia latifasciata*	Differential expression of 104 miRNAs both up-and down-regulated and targeted at the brain, muscle and gonads were identified in the cichlid fish harboring Bs. The probably associated function with differentially expressed miRNA targets in Bs possessing samples included nuclear matrix organization and response to stimuli	Nascimento-Oliveira et al. (2021)
*A. scabripinnis, A. paranae, A bockmanni, and A. fasciatus*	Protein-coding gene content of the B chromosomes in *Astyanax* sp was analyzed utilizing comparative genomic and transcriptomic Illumina data between B+ and B- individuals. Results showed sharing of protein-coding genes by Bs in the four analyzed species. 80% of the B-specific transcripts found in ovaries belonged to the oogenesis regulating gene nobox that showed >30 times expression in B when compared to A. This supports the long-term survival of B chromosomes in the population leading to speciation	Silva et al. (2021)
*A. latifasciata*	Bs from *A. latifasciata* were analyzed to reveal numerous repetitive DNA sequences, protein-coding genes and sex-biased effects. In addition, cell cycle genes such as *separin*-like, *kif11*-like and *tubb1*-like were identified. Further, proteins SMC3, SYCP1 and SYCP3 were identified that aid the self-pairing of Bs suggesting isochromosome formation was a valid step during the evolution of Bs	Cardoso et al. (2022)
*Apodemus flavicollis*	Bs from *A. flavicollis* were analyzed and the genetic content of Bs was found preserved. Differential expression of three genes *Rraga*, *Haus6*, and *Cenpe* were observed in individuals with a variable number of Bs (0–3). In addition, the accumulation of these transcripts was found to vary with the age of the animal	Rajičić et al. (2022)
*Psalidodon scabripinnis*	Reviewed available information on Bs in the *P. scabripinnis* species. In addition, they also proposed a novel chromosome speciation model that is rather facilitated by the Bs	Silva et al. (2022)

^a^ Reports collated between the years 2020 and 2022.

Molecular cytogenetics identified rRNA genes in *S. cereale* (Rimpau and Flavell, 1975), *Brachyscome dichromosomatica* (Donald et al., 1995), *C. capillaris* (Leach et al., 2005), *Trichogramma kaykai* (van Vugt et al., 2005), *E. plorans* (Ruiz-Estévez et al., 2014) and *Astatotilapia latifasciata* (Poletto et al., 2010). A few histones and SnRNA genes, some inactive ribosomal genes in raccoon dogs, C-KIT gene in mammals (*Vulpes vulpes* and *Nyctereutes procyonoides*) are reported on Bs (Szczerbal and Switonski, 2003; Teruel et al., 2010; Becker et al., 2011; Ruiz-Estévez et al., 2014; Silva et al., 2014; Marques et al., 2018). Also, transposable elements, satellite DNA, genes from multigene families such as H1, H3 and H4 histones have also been recognized in Bs (Valente et al., 2017). In *Cestrum* spp.*,* multiple types of repetitive DNA were identified in A and B chromosomes- such as 35S and 5S rDNA, AT-rich SSR, retrotransposons, TR, SINEs (short interspersed nuclear elements), LINEs (long interspersed nuclear elements) and interstitial telomeric sequences, ribosomal DNA clusters or histone genes (Ostromyshenskii et al., 2018).

The first transcriptionally active genes (*TNNI3K*, *FPGT* and *LRRIQ3*) were reported on Bs of protein-coding *Pygargus* (Siberian roe deer) followed by active genes for antibiotic and fungal resistance in *N. haematococca* and *A. sativa* (Miao et al., 1991; Dherawattana and Sadanaga, 1973). Transcriptionally active B-enriched repetitive sequences and retrotransposon-derived high-copy elements were also reported in maize and rye, a few of which were active in a tissue-dependent manner (Carchilan et al., 2007; Klemme et al., 2013). In a comparative analysis of B+ and B- genome of *L. rubripinnis* cichlid (Yoshida et al., 2011), five protein coding genes were identified out of which one gene *IHHB* was selectively and extensively amplified in the B+ individual's genome.

B chromosomes behaving as a sex chromosome with male sex-determining genes in *A. mexicanus* and in *D. albomicans* males along with inbred female flies provided an unprecedented connection between the births of the Y chromosome and origin of B (Zhou et al., 2012; Imarazene et al., 2021). In cichlid species *L. rubripinnis*, female-specific B chromosomes were observed. Further, cross-breeding experiments employing females with and without B chromosomes provided evidence that five protein-coding genes exist on the B chromosome of these species to govern female sex determination (Yoshida et al., 2011). Several B-linked genes were identified to be involved in cellular processes like microtubule organization (*TUBBI, TUBB5*), recombination (*XRCC2, SYCP2, RTELI*), kinetochore structure (*SKA1, KIF11, CEN-E*), cell cycle progression (*Separase, AURK*) (25). B chromosomes in reptilian species *Anolis cardinensis*, Chinese raccoon dog, and red fox showed genes for cell cycle and neuron synapses (Kichigin et al., 2019). In order to understand the expression of genes located on B, recently, integrative genomics and transcriptomic approach is used. In the rye plant, nine pseudogenic transcripts and active copies of an Argonaute–like *AGO4B* gene were located. A total of six fragmented and four intact genes were localized on Bs of *E. plorans*. On comparing the expression of six fragmented genes between B+ and B- individuals of *E. plorans,* five of them (*CIP2A, CKAP, CAP-G, KIF2OA and MYCB2*) were actively transcribed and upregulated in B+ organisms. In *Z. mays*, a few genes involved in nucleotide binding and cell metabolism were found to be upregulated (Huang et al., 2016). A combined approach of Illumina and Pacbio sequencing identified many genes fragment on Bs, involved in regulating the establishment of Bs in *Cichlids* species from Lake Malawi*.* Microdissection of Bs, their sequencing, assembly and annotation reported 75 genes in Asian Seabass, out of which expression of 10 (*ASTN2, FBXO33, FKBP, BRE, DPF3, GABRB2, MYT1L, RAB14, RXRAB and PACRG*) were localized in brain and gonads (Komissarov et al., 2018). A recent comparative account of B+ and B- transcriptome in *E. plorans* showed differential expression of 46 genes involved in functions like histone methyl transferase activity, protein modification, gene regulation, cell death, stress response, and chemical defense (Navarro-Domínguezet al., 2019). Complete genome sequencing in two fishes *A. mexicanus and A. correntinus* and a grasshopper species *Abracis flavolineta* localized genes and repeat content on their Bs. Gene annotation of the sequences showed the presence of some novel intact coding genes for metabolism, morphogenesis, reproduction, transposition, recombination and cell cycle imparting evolutionary success in these organisms (Ahmad et al., 2020). The paternal sex ratio (PSR) chromosome, a B chromosome in *N. vitripennis* (Jewel wasp) show the male drive following conversion of female to male by destryoing the sperm’s heredity material in young diploid embryos to. RNA interference (RNAi) technique showed that, “*haplodizer*”, a PSR-linked gene expressed in testis, codes for a putative DNA binding protein that specifically binds to sperm chromatin and eliminates it (Dalla Benetta et al., 2020).

In another study in *A. latifasciata,* cell cycle genes, *separin, tubb1* and *kif11* genes were reported which code for synaptonemal complex organization proteins *(*SMC3, SYCP1 and SYCP3*)* with localized transcription and expression in the encephalon, muscle and gonads. They have beneficial effects on hosts and contribute to the maintenance of Bs (Cardoso et al., 2022). In *Lilium callosum*, when the number of Bs exceeds one, both the pollen and seed fertility decrease significantly (Kimura and Kayano, 1961). While in rye, expression of heat stress-related genes *Hsp101*, *E3900* and *tE3900* (truncated) was about three-fold in 0B plants and about 40-four-fold upregulated in 2B plants at the pachytene stage of meiosis (Pereira et al., 2017).

Bs of the fungi *M. oryzae* and *Z. tritici* carry virulence-resistant genes which contribute to their evolution (Habig et al., 2017; Langner et al., 2021). Several phenotypic characters are regulated by B genes, for instance, sex determination in cichlid fishes (Yoshida et al., 2011) and frog *L. hochstetteri*, achene color in *Haplopappus gracilis* (Jackson and Newmark, 1960), striping of leaf in maize (Staub, 1987), and crown rust resistance in *A. sativa* (Dherawattana and Sadanaga, 1973) are reported as an outcome of the cumulative effect of Bs, depending on their total number in a cell and not merely on their presence or absence (Carlson, 2009).

Transcriptome analysis provides novel insights into the intricate interrelationship between A- and supernumerary B chromosomes as seen in an oat-maize addition line wherein Starter + B is transcriptionally active and its presence alters the maize transcriptome (Huang et al., 2016). Lately, Karafiátová et al. (2021) revealed a size of 421 Mb for the sequenced B chromosome in *Sorghum purpureosericeum* through scattered shotgun sequencing of the B chromosome. They unleashed B-specific markers in the form of nine putative B-specific repeat clusters of which SpuCL168 and SpuCL115 exclusively hybridized to centromeric regions as shown by FISH analysis. The recent high-quality sequencing of the B chromosome of maize (Blavet et al., 2021) has revealed 758 predicted protein-encoding genes, many showing known functions and some possibly helping in the perpetuation of Bs.

The latest B-omics research has revealed a lot of novel information about differentially expressed genes and proteins on the B chromosomes. The RNA-seq analysis of leaf tissues from plants with and without Bs in *Lilium amabile* showed differential expression of 5.1% of total transcripts in B-containing plants (Park et al., 2019). Notably, 4,059 (52%) differentially expressed genes (DEG), were upregulated. The functional enrichment analysis assigned important cellular functions like chromosome breakage and repair, microtubule formation, cell cycle, etc. to the upregulated genes confirming that 50% of the Bs genome is directed towards their maintenance and perpetuation (Park et al., 2019). Further, Silva et al. (2021) revealed differential transcriptomes between 0B and 1B individuals of *A. scrabripinnis* and *A. paranae* and found that the B chromosome of these two species shared 19-protein coding genes. About 80% of the B-derived ovarian transcripts belonged to an oogenesis regulatory gene *nobox*, which is 30 times upregulated in Bs as compared to its A paralogue. This altered expression of the gene *nobox* in B-carrying females was suggested to be the key mechanism in B-transmission. Recently, Boudichevskaia et al. (2022) conducted a cDNA AFLP analysis followed by a comparative RNA-seq analysis to investigate the transcriptomes of B+ and B- anthers in wheat and rye. The study revealed that rye Bs influence the expression of genes encoded by A chromosome and cellular processes like chromatin organization, gene silencing and epigenetic processes such as DNA methylation and demethylation and post-embryonic development. Additionally, it was observed that 5–6% of the standard A-derived transcripts in wheat and rye were affected in the presence of 2Bs of rye.

Furthermore, Shi et al. (2022) analyzed the global expression of genes, miRNAs and transposable elements (TEs) in maize plants with 0–7 Bs and showed the presence of active genes on maize Bs that influenced the expression of genes on As. The presence of Bs resulted in differential expression of more than 3,000 A chromosome genes and increased expression of A-located miRNAs. Interestingly, while the mere presence of B chromosomes modulated the A genes’ expression, for the B-located genes, a gene dosage effect that positively correlated with the B copy number was observed (Shi et al., 2022). The above comprehensive reports showcase the long journey endured by B chromosomes from being considered selfish to becoming unselfish and performing such important functions in the cell.

## 10 Conclusion and future perspective

The availability of the sequence information of the Bs from different organisms using the next-generation cutting-edge sequencing technologies has highlighted new roles for these hitherto “supernumerary” chromosomes. Bs have been observed to be a unique assembly of various genomic fractions including the euchromatic, heterochromatic and organellar regions pseudogenes and transposable elements that might serve as “SOS backup genomic reservoirs” of the cell that can be co-opted to shell out genic sequences and perform an array of cellular functions as and when required. The recent findings of many transcriptionally active B-linked genes disapprove of their genetic inert nature. Further, allocation of functions like sex determination, involvement in cell division and cell cycle, development, ion transport, metabolism, and regulation of gene expression of A chromosomes indicates towards Bs performing functions beyond their self-sustenance and becoming an integral part of the genome in the form of sex chromosomes or germline restricted chromosomes.

The study of Bs assembly has great evolutionary significance and Bs can serve as model systems to study the mechanisms of rapid genomic changes, construction of artificial chromosomes, manipulation of crops, as experimental tools in cancer related research and many other areas of biology. The extensive dynamism shown by Bs in their distribution, structure, function and evolution is probably due to a low selection pressure exerted on them owing to their non-essential nature. The differential presence of Bs in related individuals and populations and their correlation with both negative and positive consequences on the host has intrigued biologists. In the instances with no drive mechanism, Bs have aligned more strongly with the geographical distribution, conferring reproductive and adaptive significance to the host and leading to the fitness of populations in specific conditions. Without such functions, Bs would probably be lost unless they are positively selected by nature for some beneficial advantage.

In nutshell, the evidence suggests that the interaction between these accessory entities, the reproductive strategy of a population, and its inhabited geographical environment might determine if Bs are retained and reinforce adaptive significance or not. The number of Bs possessed ultimately seems to be the outcome of a trade-off between the chromosome drive and/or the adaptive significance and the deleterious effects of Bs on the fitness of a population.

Although, the availability of the Bs sequence has given insights into their molecular composition and functionality, many questions related to the mechanism of drive and non-disjunction process, preferential fertilization of sperms carrying non-disjoined chromosomes, unique stability and transmission mechanism of B univalent, and dodging of recombination process to bring about crossing over in the heterochromatin regions of Bs to ensure the segregation and transmission of paired Bs remain unanswered. Further, their differential presence among taxa and organs within the same organism and Bs-mediated epigenetic regulation of A chromosomal gene functions intrigue equally and are interesting avenues to explore. Future research involving advanced molecular cytogenetics, genomics, and transcriptomics studies in B chromosome mutants with and without drives will hopefully help in getting to the bottom of these perplexing mysteries that have defied explanations and baffled biologists for years. The targets for drivers are anticipated to be genes or the repeat sequence (satDNA) that are abundantly found on Bs. Since most of the genes are paralogous copies of the As, the pseudogenized sequences that are sources of endogenous siRNAs that may influence the gene expression can be the other plausible pathway that can be investigated. Since most of the observations on Bs are still incidental, the extant quest continues for the identification of the genetic and/or epigenetic system steering the drive process that propels Bs to the next generations.

## Glossary

Argonaute like-AGO4B: a group of proteins involved in both transcriptional as well as post-transcriptional gene silencing. They bind to micro or short interfering RNAs and mediate repression of specific target RNAs either by RNA degradation or by inhibiting translation.
Centromere: a region of highly specialized repetitive chromatin which mediates equal distribution of the duplicated genomes between two daughter cells. This function is achieved by recruiting a kinetochore protein complex that orients the replicated chromosome pairs to the mitotic or meiotic spindle structure.
CenH3: cenH3 is a centromere-specific histone variant, which replaces the canonical H3 in the centromeric nucleosome. It is required for kinetochore formation, mitotic progression and chromosome segregation.
Centric fragments: the fragment of a chromosome that contains a centromere dividing the chromosome into two distinct areas.
Centromeric histone variant: these are variant histone proteins that substitute for the core canonical histones (H3, H4, H2A, H2B) in nucleosomes of eukaryotes and often confer specific structural and functional features.
Drive mechanism: a mechanism through which B chromosomes gain an advantage in transmission by exceeding the normal rate during cell division
DNA amplification: It is defined as the production of multiple copies of a sequence of that can occur naturally in a living cell or can be carried out *in vitro*.
Duplication: the amplification of copy number of DNA sequences which results in an evolutionary advantage for a genome.
histone H3K27me3: it is a modified histone involved in gene silencing. It silences the expression of important developmental genes during embryonic stem cell differentiation.
Isochromosomes: the chromosomesthat have theirboth arms identical.
Introgression: the transfer of genetic information from one species to another as a result of hybridization between them and repeated backcrossing.
Mini B chromosomes: these are small chromatin segments that resemble a chromosome and consist of centromeres, telomeres and replication origins. They are created by natural structural rearrangements and transposition events occurring in normal chromosomes
Male drive: it is a drive mechanism in the B chromosome that involves non-disjunction at the second pollen mitosis producing two sperm cells.The B-containing sperm cell then preferentially fertilizes the egg in the process of double fertilization.
Non-disjunction: it is the process where both sister chromatids or homologous chromosomes are pulled to one pole of the cell during anaphase of mitosis or anaphase I and II of meiosis
Pericentromere: it is the heterochromatic region flanking the centromeric domain. The region is made up of highly methylated DNA showing more regular nucleosome spacing.Together with the centromere, it regulates accurate segregation during cell division across eukaryotes
Pycnotic cycle: During cell division chromatin undergoesa cyclic condensed state to facilitate segregation of chromosome and a decondensed state for DNA replication which is referred as pycnotic cycle of condensation–decondensation.
Preferential segregation: it is the unequal segregation of B chromosomes in gametes that is preferred during fertilization to ensure their successful transmission in the progeny
Proto B: it is the initial unstable form of B chromosome that arise in the beginning of the evolutionary process and later gets stabilized by escaping through a drive mechanism.
Paternal sex ratio (PSR) chromosome: a type of supernumerary chromosomethatoccurs in haplodiploid arthropods and is transmitted through sperm. This results in a loss of the paternal chromosomes during the early development of a diploid fertilized egg into the haploid male.
Relaxed purifying selection: it is a gene-wideor genome-wide reduction in the efficiency or intensity of purifying selection allowing exploration of a wider subset of phenotypic space leading to evolutionary processes.
satDNA: refers to a very large array of tandemly repeating, non-coding DNA. It is a structural constituent of heterochromatin and an important component of functional centromeres.
Transposition: it is the movement of DNA segments known as transposable elements from one region to the other in the genome. This mechanism is considered to be one of the most significant earliest events in the evolution of Bs.
